# Calcium Signalling in Alzheimer’s Disease: From Pathophysiological Regulation to Therapeutic Approaches

**DOI:** 10.3390/cells10010140

**Published:** 2021-01-12

**Authors:** Mounia Chami

**Affiliations:** Laboratory of Excellence DistALZ, INSERM, CNRS, Institute of Molecular and Cellular Pharmacology, Université Côte d’Azur, Sophia-Antipolis, 06560 Valbonne, France; mchami@ipmc.cnrs.fr; Tel.: +33-4939-53453; Fax: +33-4939-53408

Alzheimer’s disease (AD) is a neurodegenerative pathology representing a socioeconomic challenge, however, the complex mechanism behind the disease is not yet fully understood. AD is commonly defined as a proteinopathy characterized by the accumulation of intracellular neurofibrillary tangles composed of abnormal hyper-phosphorylated, conformated, and truncated tau, as well as extracellular deposits of β-amyloid (Aβ) species forming amyloid plaques in different brain areas [1]. The “amyloidogenic hypothesis” in AD postulates that the accumulation of Aβ plaques acts as a pathological trigger for a cascade that includes neuritic injury, the formation of neurofibrillary tangles via tau protein leading to neuronal dysfunction, and cell death [2]. This hypothesis is supported by genetic, biochemical, and pathological evidence linking familial autosomal dominant mutations in the amyloid precursor protein (APP) and presenilins (PS1 and PS2) genes, triggering an imbalance between Aβ peptide production and clearance and causing early-onset neurodegeneration [3,4]. The main progress in understanding AD pathophysiology was achieved thanks to the identification of disease-causing mutations [3,4,5]. Then, the generation of cellular and mouse models expressing disease-causing genes mimicking the development of familial forms of AD (FAD) (https://www.alzforum.org/research-models/alzheimers-disease) enabled the formulation of several interconnected mechanistic theories. Among others, the “calcium hypothesis” emerged as a key AD pathogenic pathway, impacting most, if not all, cellular components of the nervous system comprising neurons and glial cells [6,7,8]. As a second messenger, calcium is critical for proper neuronal synaptic plasticity, governing learning and memory functions [9,10], and commonly described as among the major features characterizing AD [8]. The complexity of the “calcium hypothesis” relies on the fact that disturbances of calcium homeostasis affect different cellular compartments, such as mitochondria, endoplasmic reticulum (ER), lysosomes, and several microdomains within the plasma membrane, occurring through broad interventions of calcium signalling “tool-kits” (receptors, channels, binding protein, etc.). The significance of the “calcium hypothesis” in AD pathogenesis has been formally approved since calcium dyshomeostasis was reported in presymptomatic FAD study mice and thus seemed to occur prior to the development of histopathological markers or clinical symptoms. Noteworthily, disturbances of calcium signalling, largely reported in FAD study models (in vitro and in vivo) [8,11,12,13,14,15,16], were also observed in human-derived post-mortem brains [17] and fibroblasts [18,19,20], as well as recently in human-induced neurons [21,22]. 

In this Special Issue in *Cells*, six reviews address the newest results and advances in calcium signalling deregulation mechanisms in AD, how they are linked to other molecular players involved in AD pathogenesis, and the potential therapeutic approaches to correct calcium alterations to treat AD [23,24,25,26,27,28]. 

In the review by John McDaid et al. [26], the authors describe the role of calcium dysregulation in synaptic network dysfunctions in AD. The review focuses on the mechanisms impacting plasma membrane *N*-methyl-d-aspartate receptor (NMDAR), the L voltage-gated calcium channel (VGCC), and the nicotinic acetylcholine receptor (α7nAchR) function [26]. The discussed data draw upon the complexity of calcium-synaptic dysfunction connections observed in AD mice models. The authors point out the role of extracellular Aβ plaques and toxic soluble Aβ oligomers towards synaptic hyperactivity and highlight studies demonstrating the contribution of intracellular tau to synaptic loss and the impairment of synaptic function. They also provide evidence demonstrating that synaptic plasticity dysfunctions in AD are linked to excessive ER calcium release, mainly through the ryanodine receptor (RyR) by the process of calcium-induced calcium release (CICR). In addition, the review by John McDaid et al. provides key elements demonstrating the role of calcium dyshomeostasis in lysosome-autophagosome-mediated protein degradation in AD [26]. Noteworthily, enhanced lysosomal calcium efflux is seen as an early event in AD pathology, contributing to defective lysosome—autophagy degradative function but also to synaptic transmission deficiency [29].

In addition to synaptic plasticity deficits, calcium dyshomeostasis has a profound effect on the function of cell organelles, including ER and mitochondria, both of which play an important role in maintaining cellular and synaptic function. These specific items were discussed in our review [23] and in that by Noemi Esteras and Andrey Y. Abramov [25], respectively. 

In our review [23], we describe the main neuronal calcium signalling “tool-kits” and focus on ER calcium handling molecules alterations in AD and the benefit of targeting the aforementioned to alleviate AD pathogenesis. Our review describes the tight link between the “calcium hypothesis” and the amyloidogenic cascade generating Aβ peptides and other APP-derived toxic fragments [30]. ER calcium mishandling in AD includes alterations of the inositol 1,4,5-trisphosphatereceptors (IP_3_Rs) and ryanodine receptors (RyRs) expression and function, the dysfunction of the sarco-endoplasmic reticulum calcium ATPase (SERCA) activity, and the upregulation of SERCA1 truncated isoform (S1T), as well as presenilins (PS1, PS2), forming the catalytic core of the γ-secretase enzymatic complex cleaving APP [23]. We summarize the neuronal expression, structure, and physiological function for each ER molecular component. The sum of studies discussed offers an outline of the disease-associated remodelling of ER calcium machinery coupled to specific cellular signalling cascades modulating the activity (i.e., post-translational modifications, interactions with regulatory proteins) and/or the expression of ER calcium channels and pump. The depletion of ER calcium content activates the store-operated calcium entry (SOCE) pathway [31]. We then report studies describing the expression and function alterations of the molecular bridge linking ER calcium depletion and the activation of plasma membrane calcium entry implicating STIM and ORAI proteins [23]. 

The review by Noemi Esteras and Andrey Y. Abramov specifically describes the mechanisms underlying mitochondrial calcium deregulation linked to Aβ and tau pathologies [25]. They first depict the basis of physiological mitochondrial calcium homeostasis and then describe the cytosolic and mitochondrial calcium homeostasis impairments in AD and in tauopathies (neurodegenerative disorders characterized by the deposition of abnormal tau protein in the brain) [25]. The authors specifically discuss the molecular mechanisms underlying mitochondrial calcium disturbances and expose complementary scenarios linking the deleterious mitochondria calcium overload to neuronal death [25]. These mechanisms include the alteration of the expression of mitochondrial calcium-related proteins and of ER–mitochondria interactions, and also the impairment of mitochondrial calcium efflux, and mitochondrial permeability transition pore opening. These mechanisms appear to act in concert in the process of neurodegeneration in AD and tauopathies [25]. 

In addition to forming the catalytic core of the γ-secretase enzyme, several studies have demonstrated a role of PS1 and PS2 in subcellular calcium signalling. Our review [23] and that by John McDaid et al. [26] extensively highlight the role of PS1 in controlling several aspects of the subcellular calcium signalling deregulation and in synaptic plasticity. The review by Paola Pizzo et al. [28] specifically focuses on the role of PS2 in the modulation of ER and Golgi apparatus calcium handling, calcium entry through the plasma membrane channels, mitochondrial function, ER–mitochondria communication, and autophagy. The authors overview the alterations of calcium homeostasis observed in several cell lines expressing FAD-PS2 mutants, in human-derived fibroblasts, and in PS2 mice and ex vivo models (primary neurons culture and acute hippocampal slices) [28]. Of most interest, they discuss the impact of familial PS2 mutations in the control of multiple aspects of cell and tissue physiology, including cell metabolism and bioenergetic and brain network excitability [28]. 

The review by Veronika Prikhodko et al. [27] focuses on the TRPC6 (transient receptor potential channel 6), a non-selective cation plasma membrane channel that is permeable to calcium and activated by the emptying of the ER calcium store in a SOCE-dependent manner [32]. The review describes the role of TRPC6 in AD and brain ischemia [33,34]. The authors argue that although the pathophysiological mechanisms causing AD and cerebral ischemia may differ, cerebral ischemia serves as a risk factor for AD development, and vice versa. They postulate that both pathologies share a common mechanism associated with intracellular calcium dyshomeostasis likely implicating TRPC6. The review describes the contribution of the TRPC6 in neuronal hypo- or hyper-activation in both pathologies, with a particular focus on calcium entry alteration. The authors then discuss the potential drug candidates targeting TRPC6 that have shown some beneficial therapeutic effects in different cellular and animal models [27]. 

The review by Maria Calvo-Rodriguez et al. [24] describes AD-related calcium disturbances in neurons, astrocytes, and microglia. The authors discuss studies demonstrating that enhanced cytosolic calcium levels linked to Aβ and also to APOE4 (a genetic risk factor for sporadic AD forms) likely contribute to astrogliosis [35]. Importantly, the enhanced frequency of spontaneous calcium waves and calcium hyperactivity in astrocytes were observed in the intact brain of AD mice. The authors also report that calcium homeostasis was impaired in microglia isolated from AD mice, likely contributing to their activation. In a specific section, the authors highlight studies using intravital imaging to directly monitor the cytosolic calcium content in transgenic AD mice brains [24]. The review is composed of different chapters describing the contribution and the potential therapeutic effect of distinct calcium channels of the plasma membrane, endoplasmic reticulum, SOCE, mitochondria, and lysosomes [24]. The authors discuss the available therapeutic strategies targeting Aβ and emphasize the potential benefits in the genetic and immunomodulation of tau, and review the different strategies for targeting calcium deregulation, such as therapeutics in AD including human data and those generated from experimental models. 

To conclude, this Special Issue provides recent research insights in the field of calcium signalling involvement in AD, which may open new research hypotheses and stimulate the development of therapeutic strategies.
